# Conception Rate and Reproductive Hormone Secretion in Holstein Cows Immunized against Inhibin and Subjected to the Ovsynch Protocol

**DOI:** 10.3390/ani10020313

**Published:** 2020-02-17

**Authors:** Rihong Guo, Fang Chen, Cheng Mei, Zicun Dai, Leyan Yan, Zhendan Shi

**Affiliations:** 1 Institute of Animal Science, Jiangsu Academy of Agricultural Sciences, Nanjing 210014, China; rhguo@jaas.ac.cn (R.G.); fchen_m@sina.com (F.C.);; 2 Dongying Austasia Modern Dairy Farm Co., Ltd., Dongying 257345, China; cheng.mei@japfa.com

**Keywords:** inhibin, immunization, conception rate, hormone, Holstein cows

## Abstract

**Simple Summary:**

In recent decades, conception rates in lactating dairy cows have dramatically decreased, and improving the conception rate has become a major challenge in dairy cow reproduction. Various Ovsynch protocols have been developed to aid in the resumption of ovarian follicular activity for timely breeding in post-partum cows. However, the effect of Ovsynch protocols on improving the conception rate is unsatisfactory. In addition, immunization against inhibin was reported to improve the fertility of domestic animals. Thus, a novel reproductive technique combining immunization against inhibin and the widely used Ovsynch protocol was proposed and tested in this study. Our results showed that immunization against inhibin has the potential to improve conception rates in cows, but also compromised luteal function. According to these results, additional luteal-stimulating treatments are suggested to further improve cow fertility based on immunization and the Ovsynch protocol.

**Abstract:**

This study was conducted to investigate the feasibility of improving fertility in dairy cows via immunization against inhibin. Thirty-two cows were divided into Control (n = 11), Low-dose (n = 10) and High-dose (n = 11) groups. The High-dose and Low-dose cows were treated with 1 and 0.5 mg of the inhibin immunogen, respectively. All the cows were subjected to the Ovsynch protocol from the day of antigen administration and were artificially inseminated. Blood samples were serially collected over a 24-day period from the start of the Ovsynch protocol to 14 days after insemination. The results showed that immunization against inhibin dose-dependently increased the plasma concentrations of follicle-stimulating hormone (FSH), estradiol (E2), and activin A, but decreased progesterone (P4) concentrations in the luteal phase. Immunization also increased the plasma interferon (IFN)-τ concentrations in pregnant cows on day 14 after initial insemination. The conception rates in High-dose (45.5%) and Low-dose (40%) cows marginally increased compared to that in Control cows (27.3%), but the increases were not significant (*p* > 0.05). In conclusion, a single immunization against inhibin has the potential to improve conception rates, despite impaired luteal development. To further improve the reproductive performance of dairy cows, additional luteal-stimulating treatments are suggested in combination with immunization against inhibin and Ovsynch techniques.

## 1. Introduction

Progress in dairy breeding and the application of novel technologies has dramatically increased per-cow milk yields over the past decades [1]. However, this progress has negatively affected the reproductive efficiency of lactating cows by decreasing the rate of established successful conceptions from more than 50% in the 1950s [1,2,3] to only approximately 35% recently [4], and even to approximately 25% in summer and autumn [5,6]. Improving the conception rate has become a major challenge in dairy cow reproduction. In recent decades, the Ovsynch or timed artificial insemination (TAI) technique, using gonadotropin-releasing hormone (GnRH) and prostaglandin hormones, has been widely used to aid post-partum cows to resume ovarian follicular activity for timely breeding [7].

Female fertility is determined by several factors including follicle size, oocyte quality, and embryo quality [1,8], as well as timely growth of the corpus luteum (CL), which aids in conditioning the uterus for successful embryo implantation [9]. Studies have indicated that the size of the ovulating follicle is the prime factor in determining post-insemination conception [10,11,12,13]. Fully-developed, large follicles secrete copious estradiol (E2), which is responsible for proper estrous behavior and thus for timely insemination [10,11,14]. Furthermore, a large healthy follicle can be transformed into a good-quality CL for the proper secretion of progesterone, which is essential for embryonic development and implantation [10,11,14]. However, the widely used technique to improve the reproductive performance of cows, the Ovsynch or TAI protocol, only regulates the ovarian follicular cycle, rather than enhancing follicular development in terms of quality and size, and does not enhance the quality of luteal development. Therefore, conception rates following an Ovsynch protocol or an even more complicated pre-synch or double-synch protocol remain unsatisfactory [15,16], especially during the summer heat stress period, when follicular development and oocyte quality are very poor [9,17,18,19].

Therefore, new reproductive techniques to enhance follicular development and oocyte quality are needed to improve the reproductive performance of cows. Our previous studies on improving embryo production in cows using a conventional superovulation protocol demonstrated that immunization against inhibin enhances the quality of follicular development by increasing E2 secretion and improves embryo quality and quantity [20,21,22]. In vitro studies have demonstrated that treatment with an anti-inhibin α-subunit antibody improves the quality and development of cultured bovine or porcine granulosa cells [23,24,25], with substantial enhancements in E2 secretion, cell proliferation, and the expression levels of genes involved in follicle development and maturation [23]. In addition, the anti-inhibin α-subunit antibody treatment of in vitro-matured oocytes also improves the maturation rate and subsequently, the quality of early embryo development [21,22,26]. These in vivo and in vitro results indicated that immunization against inhibin could enhance ovarian follicle, oocyte, and early embryo development. Therefore, this study aimed to determine the feasibility of improving conception rates or the reproductive performance of cows using immunization against inhibin.

## 2. Materials and Methods

### 2.1. Immunogen

The immunogen was prepared as previously reported [26]. Briefly, recombinant porcine inhibin α-subunit was expressed in bacteria transfected with the expression vector pRSETA-INHα. Then, this recombinant protein was purified and homogenized with mineral oil adjuvant to reach a final concentration of 1 mg/mL for use as an immunogen in the experiment. The placebo immunogen was prepared with physiological saline homogenized with mineral oil adjuvant.

### 2.2. Animals and Treatments

**Figure 1:**
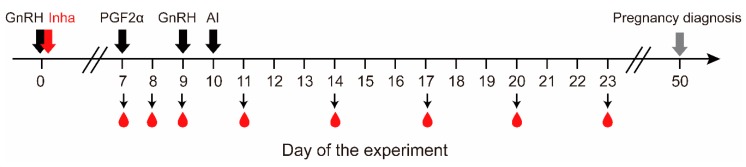
An outline explaining the experimental design. The wide black arrows indicate Ovsynch procedures involving two injections of GnRH and one injection of PGF2α; the wide red arrow indicates immunization against inhibin α-subunit or placebo immunogen; the grey arrow indicates pregnancy diagnosis based on ultrasonography examinations; small arrows and droplets indicate blood sampling. The immunogen used was 0.5 and 1 mg of recombinant inhibin protein per cow for the Low-dose and High-dose groups, respectively.

This study was carried out at Huamei Dairy Farm in Guangdong province, Southern China. The experiment was performed from the time between late spring to summer (March–May). Thirty-two Holstein dairy cows were selected according to their body condition score, normal estrus cycles, similar ovarian follicle numbers, and presence of active CL, as determined by ultrasonography (Aloka SSD-500, Aloka Co., Ltd., Tokyo, Japan). The cows were milked twice daily, and milk production was recorded monthly. Milk yield per lactation averaged approximately 4600 kg with peak yields of 23 kg per day. The cows were 60 to 96 months of age and in the range 60–110 days postpartum. The cows were kept in a good hygienic environment with comfortable housing, supplied with fresh and clean water (free access), and fed a complete mixed ration according to the nutritional requirements (NRC) of Holstein cows. All cows underwent a conventional Ovsynch hormone treatment protocol as follows: 100 µg GnRH/head (Ningbo No.2 hormone factory, Zhejiang, China) on day 1, 0.5 mg/head PGF2α (Shanghai Institute of Planned Parenthood Research, Shanghai, China) on day 7, and 100 µg GnRH/head on day 9; insemination was carried out 16–18 h later. The experimental designs are illustrated in Figure 1.

The cows were divided into three groups, namely High-dose (n = 11), Low-dose (n = 10), and Control (n = 11) groups. On day 1 of the experiment, the High- and Low-dose groups were i.m. administered 1 mL and 0.5 mL of the immunogen with 1 mg and 0.5 inhibin α-subunit fusion protein, respectively, whereas the Control cows were treated with placebo immunogen. 

This experiment was approved by the Research Committee of Jiangsu Academy of Agricultural Sciences under the guidance of the Regulations for the Administration of Affairs Concerning Experimental Animals (Decree No.2 of the State Science and Technology Commission on November 14, 1988).

### 2.3. Blood Sampling

To measure antibody titers and plasma concentrations of follicle-stimulating hormone (FSH), E2, activin A, progesterone (P4), and interferon (IFN)-τ following immunization against inhibin, blood samples (10 mL) were collected from each cow on days 7, 8, 9, 11, 14, 17, 20, and 23 via venipuncture of the jugular vein into a tube containing 100 IU of heparin. The samples were centrifuged at 1000× *g* for 20 min within 2 h. All plasma samples were stored at −20 ℃ until analysis.

### 2.4. Measurement of Blood Antibody Titers

A standard enzyme-linked immunosorbent assay (ELISA) was adopted to measure the anti-inhibin antibody titers in plasma [27]. The recombinant inhibin was coated in 96-well microtiter plates (0.5 µg/well in 100 µL). Then, 100 µL of diluted plasma sample (1:800 dilution in 5% skim milk) was added to the wells and incubated for 1 h. The bound antibody was further labeled via incubation with horseradish peroxidase (HRP)-conjugated rabbit anti-bovine antibody (Solarbio, Beijing, China). The detection of binding was initiated by the addition of the chromogen tetramethylbenzidine (Sigma Chemical Co., St Louis, MO, USA) solution containing 0.03% H_2_O_2_ and was terminated as appropriate with the addition of 2% H_2_SO_4_. The optical absorbance at 450 nm that represents the anti-inhibin antibody titer was measured. To overcome treatment bias in assay results, plasma samples from all cows that were collected during the same collection event were measured on the same plate.

### 2.5. Measurements of Hormone Concentrations in Blood

Concentrations of plasma E2, P4, FSH, and activin A were measured as previously reported [21,27]. In brief, E2 and P4 were measured using medical diagnosis RIA kits (Beijing Northern Biotechnology Institute, Beijing, China) and FSH and activin A were detected using respective bovine ELISA kits (R&D Systems China, Shanghai, China). Plasma IFN-τ concentrations were measured using the bovine IFN-τ kit (Mlbio, Shanghai, China). The sensitivity and measurement range of the IFN-τ kit were 1 pg/mL and 7.5–240 pg/mL, respectively. The inter- and intra-assay coefficients of variation of each kit were less than 10%.

### 2.6. Statistical Analysis

Differences in the conception rates were determined using the Chi-squared test. Differences in plasma E2, FSH, P4, activin A, and IFN-τ concentrations and anti-inhibin antibody titers between the three groups were calculated using one-way analysis of variance (ANOVA), following the Tukey test for multiple comparisons. All statistical analyses were performed using IBM SPSS Statistics (SPSS Inc., Chicago, IL, USA). Data are presented as the mean ± standard error of mean (SEM), and *p <* 0.05 was considered significant.

## 3. Results

### 3.1. Effects of Inhibin Immunization on Conception Rates in Holstein Cows

The conception rates in the High-dose group (45.5%, 5/11) and Low-dose group (40.0%, 4/10) increased by 66.7% (*p* = 0.18) and 46.5% (*p* = 0.27), respectively, compared to that in Control cows (27.3%, 3/11), but these increment were not significant (Table 1).

### 3.2. Antibody Titers

**Figure 2:**
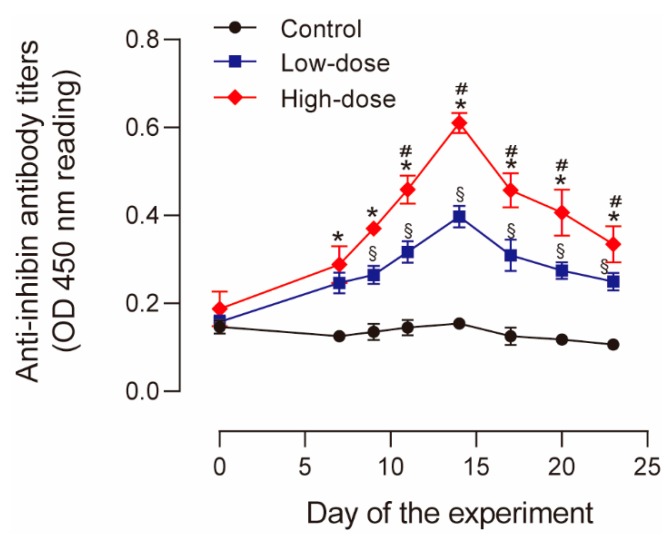
Anti-inhibin antibody titers following immunization in High-dose, Low-dose, and Control cows. Data are presented as the mean ± standard error of mean (SEM). * *p* < 0.05, High-dose group vs. Control group; # *p* < 0.05, High-dose group vs. Low-dose group; § *p* < 0.05, Low-dose group vs. Control group.

The antibody titers steadily increased in both the High-dose and Low-dose groups after immunization, and the increase became significant from day 7 and 8, respectively (Figure 2). The increase in antibody titers was relatively higher in the High-dose group compared to that in the Low-dose group, the difference of antibody titers between these two immunized groups became significant from day 11 (Figure 2). The titers in both groups peaked at day 14 and then slowly declined during the remaining time (Figure 2). Meanwhile, the antibody titers in Control cows remained at baseline levels throughout the experiment (Figure 2). These results indicated that a single immunization with both 0.5 and 1 mg of recombinant protein could efficiently increase anti-inhibin antibody titers, and the use of 1 mg immunogen is more effective.

### 3.3. Plasma Reproductive Hormone Profile

**Figure 3:**
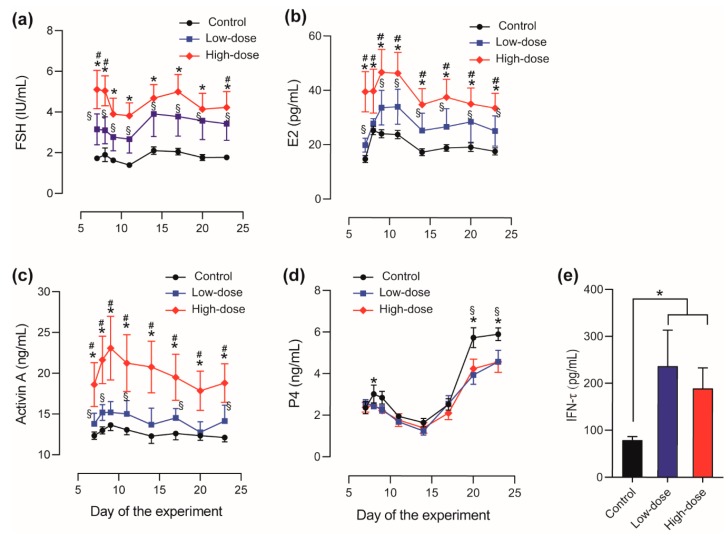
Plasma concentrations of follicle-stimulating hormone (FSH) (**a**), estradiol (E2) (**b**), activin A (**c**), progesterone (P4) (**d**), and interferon (IFN)-τ (**e**) in High-dose, Low-dose, and Control cows. Data are presented as the mean ± SEM. In (**a**)–(**d**), * *p* < 0.05, High-dose group vs. Control group; # *p* < 0.05, High-dose group vs. Low-dose group; § *p* < 0.05, Low-dose group vs. Control group. In (**e**), * *p* < 0.05 vs. the control group.

The plasma concentrations of FSH, E2, activin A, P4, and IFN-τ are presented in Figure 3.

Plasma FSH concentrations (Figure 3a) declined from day 8 to 11 in all three groups, and then started to rise from day 11, peaking on day 14 in the Low-dose and Control groups and on day 17 in the High-dose group. Subsequently, plasma FSH concentrations slightly declined in all three groups. Plasma E2 (Figure 3b) and activin A (Figure 3c) concentrations shared similar patterns, increasing from day 6 and peaking on day 9 or 11 at the time of ovulation and insemination in all three groups. Subsequently, E2 and activin A concentrations decreased to low levels on approximately day 14 and fluctuated somewhat during the remaining test period. Immunization against inhibin dose-dependently increased plasma FSH, E2, and activin A concentrations (Figure 3a–c). The increases in FSH, E2, and activin A concentrations after High-dose immunization were significant (*p* < 0.05) at all sampling points, whereas the increases in FSH, E2, and activin A concentrations after Low-dose immunization were also statically significant except day 8 for E2, and day 9, 14, and 20 for activin A. Plasma FSH concentration in High-dose group was significantly higher than that of the Low-dose group on day 7, 8 and 23, while plasma E2 and activin A concentrations in High-dose group were significantly higher than those in Low-dose group at all sampling points.

Plasma P4 concentrations declined from day 8, the day after PGF2α treatment, and reached their lowest level on day 14. Thereafter, P4 concentrations rapidly increased and remained high until the end of the experiment (Figure 3d). During the initial 14 days, plasma P4 concentrations were slightly higher in the Control group than in the two immunized groups, however, only the decrease of P4 concentration in High-dose group was significant compared to that in Control group on day 8. The differences in plasma P4 concentrations between the control group and treated groups increased on day 20 and 23 and reached statistical significance (*p* < 0.05). These results indicated that immunization against inhibin attenuates the P4 secretion in the luteal phase.

Plasma IFN-τ concentrations on day 23, which is 14 days after insemination, in pregnant cows were measured. IFN-τ concentrations in the immunized cows (236.6 and 183.3 pg/mL in the Low- and High-dose groups, respectively) were significantly higher (*p* < 0.05) than that in Control group (78.7 pg/mL). No significant differences were detected between the High- and Low-dose groups (Figure 3e). The increased IFN-τ concentrations suggested improved early blastocyst quality in treated pregnant cows.

## 4. Discussion

In the present study, we combined the technique of immunization against inhibin with traditional Ovsynch procedures to improve the conception rate of dairy cows. The results showed that a single immunization against inhibin dose-dependently increased the plasma concentrations of FSH, E2, and activin A, which are indicators of enhanced ovarian follicular development. These increases were associated with the marginal enhancements in conception rates in cows. Further, the enhanced conception rates were also associated with an increased concentration of plasma IFN-τ, indicating that immunization against inhibin also enhanced early embryonic development, which promoted the secretion of this anti-luteolytic cytokine.

The increase in conception rate after immunization against inhibin might be due to dose-dependent responses in the secretion of activin A, FSH, and E2. Inhibin is considered as a negative regulator of pituitary FSH secretion [28], and numerous studies on a variety of animal species have demonstrated that immunization against inhibin enhances the secretion of FSH [26,29,30,31]. Increased levels of FSH were found to stimulate the development of large, subordinate, and even small follicles in both dairy and water buffalo cows [21,32]. Since activin A is secreted by medium and small follicles [33,34], it is reasonable to assume that immunization against inhibin would also dose-dependently increase the plasma concentrations of activin A, as was observed in other studies [21,26,35]. Furthermore, in in vitro studies that used cultured ovarian granulosa cells, passive immunization against inhibin or treatment with anti-inhibin α-subunit antibodies dramatically stimulated E2 production [23,24], because inhibin negatively regulates granulosa cell development and function through para/autocrine activity [28,33,34]. Thus, the enhanced secretion of pituitary FSH and ovarian activin A combined with reduced inhibin bioactivity boosted ovarian follicle development and E2 secretion, especially in animals immunized with 1 mg inhibin antigen. The augmented E2 secretion strengthened estrous behavior and improved secretory conditions in the reproductive tract to favor insemination and spermatozoa transport, which facilitated the timely fertilization of ovulated eggs and contributed to the improved conception rates.

In our previous studies aimed at improving the embryo production efficiency by immunization against inhibin, not only embryo production, but also the embryo quality, was improved [20,21,26]. When oocytes mature in vitro, treatment with anti-inhibin α-subunit antibodies also significantly improves the oocyte maturation quality and the development competence of early embryos [20,22,26]. IFN-τ, a cytokine with anti-luteolytic function in bovine and ovine, is secreted by the trophectoderm of the preimplantation blastocyst [36,37,38,39]. We detected a dramatic increase in the IFN-τ concentration on day 14 post-insemination, indicating improved embryo or blastocyst development in the immunized cows. Immunoneutralization of inhibin bioactivity might increase the activin-to-inhibin or activin-to-follistatin ratio during the oocyte maturation stage [20,22,26], thus enhancing the quality of oocyte development by regulating cumulus cells. Therefore, the improved quality of embryos in inhibin-immunized cows should also contribute to enhanced conception rates.

Our results also demonstrated that immunization against inhibin significantly suppresses the secretion of P4, especially at 2 weeks post-insemination. Although better-developed ovarian follicles should naturally lead to an improved or larger CL [11], the super-developed follicles, obtained following immunization against inhibin, apparently did not result in good luteal function. This result might have been due to both a reduction in inhibin bioactivity and increased plasma activin concentrations following immunization against inhibin. The treatment of cultured marmoset luteal cells with antibodies to inhibin was found to decrease human chorionic gonadotropin (hCG)-induced progesterone secretion [40], whereas activin A has been shown to retard luteinization and inhibit progesterone production by luteinized granulosa cells [41,42,43,44]. It is obvious that immunization against inhibin compromises luteal development and functions and may exert a detrimental effect on conception, with reduced plasma progesterone concentrations. However, such detrimental effects could be offset by better-developed embryos in inhibin-immunized cows, in which conception rates were still enhanced. Another study conducted by our team indicated that the administration of hCG could increase the farrowing rate after immunization against inhibin, compared to that without luteal enhancement [45] (Guo et al., 2020, submitted).

Immunization against inhibin with 1 mg immunogen showed better stimulation effects on the secretion of FSH, E2 and activin A compared to 0.5 mg immunogen, and the inhibition effects and stimulation effects of immunization against inhibin on P4 and IFN-τ secretion were almost the same between the immunized groups. To this extent, 1 mg immunogen is more effective in stimulating cow reproduction performance. This is in agreement with the relatively higher increase in conception rates in the High-dose group than the Low-dose group. However, due to the limited number of cows used in this study (only about 10 each group), the increments in conception rates in both immunization groups doses didn’t reach significance. In addition, whether immunization against inhibin with a higher dose of immunogen, such as 2 mg recombinant inhibin protein, would further improve conception rates is unclear. To overcome the limitations of the current study, more cows should be used to evaluate the effects of immunization against inhibin with various doses of immunogen, such as 2 mg, 1 mg and 0.5 mg of recombinant inhibin protein, on conception rates and hormone profiles in future studies.

## 5. Conclusions

Our results suggest that a single immunization against inhibin may have the potential to improve the conception rate in cows; moreover, it dose-dependently stimulated plasma concentrations of FSH, E2, and activin A. However, immunization against inhibin negatively regulated P4 secretion, and thus, the potential to improve reproductive performance via immunization against inhibin might be limited by the side effect of impaired luteal function. To further improve the conception rate in dairy cows, luteal enhancement techniques such as post-insemination hCG administration should be incorporated into the current techniques of immunization against inhibin and the Ovsynch protocol in future studies.

## Figures and Tables

**Table 1:** Conception rates in inhibin-Immunized and Control Holstein cows.

Group *	Conception Rate (no. of Pregnant Cows)	*p*-Value
Control (n = 11)	27.3% (3)	-
Low-dose (n = 10)	40.0% (4)	0.27
High-dose (n = 11)	45.5% (5)	0.18

* High-dose and Low-dose immunized cows were i.m. administrated 1 and 0.5 mg of the recombinant inhibin α-subunit fusion protein, respectively, whereas the Control cows were given the placebo immunogen.

## Data Availability

The data that support the findings of this study are available on request from the corresponding author. The data are not publicly available due to privacy or ethical restrictions.
